# Fine mapping and identification of candidate genes for the peach powdery mildew resistance gene *Vr3*

**DOI:** 10.1038/s41438-020-00396-9

**Published:** 2020-11-01

**Authors:** Neus Marimon, Jordi Luque, Pere Arús, Iban Eduardo

**Affiliations:** 1 Centre for Research in Agricultural Genomics (CRAG) CSIC-IRTA-UAB-UB, Campus UAB, 08193 Bellaterra, Barcelona Spain; 2 IRTA (Institut de Recerca i Tecnologia Agroalimentàries), Barcelona, Spain; 3 Plant Pathology, IRTA Cabrils, Carretera de Cabrils km 2, 08348 Cabrils, Spain

**Keywords:** Plant genetics, Plant stress responses

## Abstract

Powdery mildew is one of the major diseases of peach (*Prunus persica)*, caused by the ascomycete *Podosphaera pannosa*. Currently, it is controlled through calendar-based fungicide treatments starting at petal fall, but an alternative is to develop resistant peach varieties. Previous studies mapped a resistance gene (*Vr3*) in interspecific populations between almond (‘Texas’) and peach (‘Earlygold’). To obtain molecular markers highly linked to *Vr3* and to reduce the number of candidate genes, we fine-mapped *Vr3* to a genomic region of 270 kb with 27 annotated genes. To find evidence supporting one of these positional candidate genes as being responsible of *Vr3*, we analyzed the polymorphisms of the resequences of both parents and used near-isogenic lines (NILs) for expression analysis of the positional candidate genes in symptomatic or asymptomatic leaves. Genes differentially expressed between resistant and susceptible individuals were annotated as a Disease Resistance Protein RGA2 (*Prupe2G111700*) or an Eceriferum 1 protein involved in epicuticular wax biosynthesis (*Prupe2G112800*). Only *Prupe2G111700* contained a variant predicted to have a disruptive effect on the encoded protein, and was overexpressed in both heterozygous and homozygous individuals containing the *Vr3* almond allele, compared with susceptible individuals. This information was also useful to identify and validate molecular markers tightly linked and flanking *Vr3*. In addition, the NILs used in this work will facilitate the introgression of this gene into peach elite materials, alone or pyramided with other known resistance genes such as peach powdery mildew resistance gene *Vr2*.

## Introduction

Peach [*Prunus persica* (L.) Batsch] is one of the best characterized species among Rosaceae^1–3^ and an important stone fruit crop in temperate regions: more than 24 million tons of peaches, nectarines and flat fruits produced worldwide in 2018^4^. Most commercial peach cultivars are susceptible to different pests and diseases. One of the most important being peach powdery mildew (PPM)^5,6^, caused by the ascomycete *Podosphaera pannosa*^7^. To our knowledge, all peach commercial cultivars are susceptible PPM to a variable degree. The pathogen infects the fruits, leaves, buds, and shoots, where mycelium develops as white-grayish spots on the surface, and heavy infections on fruit and leaves may induce their premature fall^7,8^. PPM can be controlled effectively through foliar fungicide applications, applied regularly every 7–14 days during the year^9^ from prebloom to the end of harvest^5^. Recently, a predictive model for disease progress has been described^10^, which included a threshold to initiate fungicide programs at early infection set.

An environmentally safe alternative to fungicide applications is the development of resistant varieties through plant breeding^6^. Little information is currently available on breeding for resistance to pests and pathogens in stone fruit crops^11^, probably due to the length of time required to introduce genes from exotic sources in perennial plants. Two descriptions of PPM major resistance genes have been published. Pascal et al.^5,6^ described a monogenic dominant locus in linkage group 8 (G8), named *Vr2*, from the peach rootstock cultivar ‘Pamirskij 5’. In the peach cross-compatible *Prunus* species almond (*P. dulcis*), Donoso et al.^12^ mapped a monogenic powdery mildew resistance gene in G2 in two interspecific populations between almond ‘Texas’ and peach ‘Earlygold’, with the dominant resistance allele from ‘Texas’. The gene, named *Vr3*, was located in a genomic region of 2.7 cM and 1.8 Mb, where 187 genes were annotated in the peach reference genome. Other resistance sources are quantitative trait loci (QTLs) controlling PPM tolerance. Pacheco-Cruz et al.^13^ described a source of tolerance from peach ‘OroA’ in G7 that could explain up to 8% of the phenotypic variation, and several QTLs have been identified in *Prunus davidiana*^7,8^. Furthermore, Dabov^14^ found that in *Prunus ferganensis*, *Vr* and *Sr* alleles conferred high and low resistance, respectively. Moreover, a lower susceptibility to PPM in peach has also been found to be associated with the presence of leaf glands, this character having been mapped at the *E/e* locus on chromosome 7^15^.

Breeding peach cultivars resistant to PPM will be more efficient with the availability of molecular markers tightly linked to genes of resistance or based on the polymorphism responsible for the resistance. Therefore, our objectives were to fine map the *Vr3* gene responsible for PPM resistance to obtain a reduced number of candidate genes and to characterize them by analyzing the polymorphisms of parent resequences and performing an expression analysis. The outcome of this study would provide valuable information on the *Vr3* candidate genes and better markers for marker assisted selection in peach breeding programs.

## Results

### Fine mapping and identification of *Vr3* candidate genes

A total of 729 descendants derived from individuals carrying *Vr3* in heterozygosis were genotyped using two SSR markers (CPDCT044 and BPPCT004) known to include *Vr3*^12^. These were from nine populations shown in Table 1 (TxE, T1E, E2T-031, 11P15, 15P15, 19P15, 25P15, and T1BT).

**Table 1:** Individuals used in fine mapping of the *Vr3* PPM resistance gene

Year	Population type	Family code	Female parent	Male parent	Individuals	Recombinant individuals	Location
**–**	F1	MB1.37	‘Texas’	‘Earlygold’	1	**-**	Caldes de Montbui
–	F2	TxE	‘MB1.37’	‘MB1.37’	111	3	Cabrils/Gimenells
–	BC1	T1E	‘MB1.37’	‘Earlygold’	189	3	Cabrils/Gimenells
2014	BC2	E2T-031	‘Earlygold’	T1E-031	26	2	Caldes de Montbui/Mollerussa
2015	F2	TxE	‘MB1.37’	‘MB1.37’	150	5	Caldes de Montbui
2015	BC2	11P15	‘Earlygold’	T1E-031	14	1	Caldes de Montbui
2015	BC3	15P15	E2T-031-005	OP	26	11	Caldes de Montbui
2015	BC3	19P15	E2T-092-002	OP	127	3	Caldes de Montbui
2015	BC3	25P15	E2T-092-021	OP	64	1	Caldes de Montbui
2015	BC2	–	T1E-042	‘Nectatop’	4	1	Caldes de Montbui
2015	BC2	–	‘Nectatop’	T1E-03	1	1	Caldes de Montbui
2015	BC2	–	‘Sweetlove’	T1E-03	2	1	Caldes de Montbui
2016	BC2	E2T-092	‘Earlygold’	T1E-092	11	1	Caldes de Montbui/Mollerussa
2016	BC3	14P16	‘Nectatop’	E2T-092-025	22	6	Mollerussa
2016	BC3	1114	P01F002A054	E2T-092-025	28	11	Mollerussa
2017	BC3	44P17	‘Nectatop’	E2T-092-025	54	2	Gimenells
2017	BC2	51P17	‘MB1.37’	OP	218	6	Caldes de Montbui
2018	BC2	72P18	T1E-021	OP	33	2	Caldes de Montbui
2018	BC2	74P18	T1E-024	OP	12	1	Caldes de Montbui
2018	BC2	84P18	T1E-040	OP	25	3	Caldes de Montbui
2018	BC2	93P18	T1E-064	OP	259	2	Caldes de Montbui
–	BC1	T1BT	‘MB1.37’	‘Big Top’	21	1	Mollerussa

*OP* open pollination

The recombination between the two markers in 30 of these 729 individuals was observed, with 16% phenotyped as resistant and 84% as susceptible. Based on the resequences of the parental lines, we designed new molecular markers, including four SSRs, 14 Indels and four SNPs (SM Tables 1 and 2). Recombinant individuals (Table 1) were genotyped using these markers to narrow down the genomic region where *Vr3* was located. After phenotyping the recombinant individuals, and using the new genotyping information, we located *Vr3* in the region between markers Indel16912 and SNP_17184692 (Table 2), corresponding to physical positions 16,912,811 and 17,184,692, respectively.

**Table 2:**
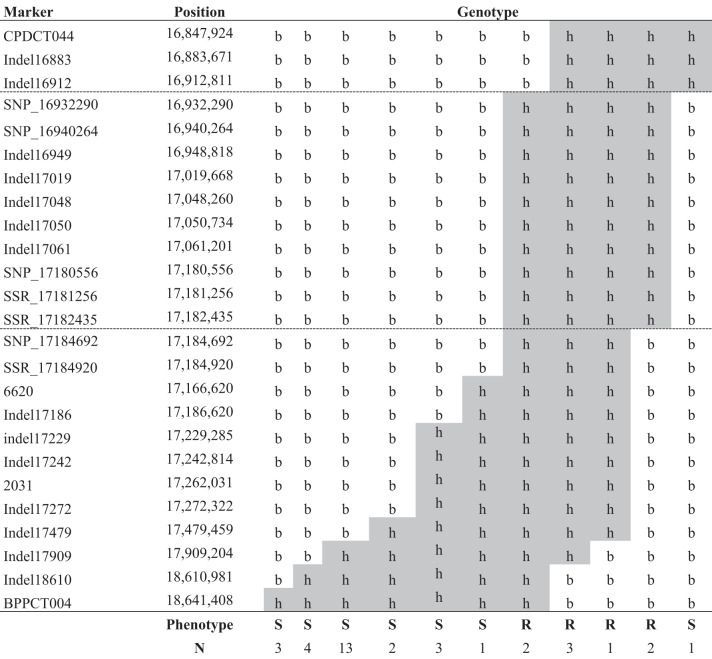
Phenotypes and genotypes of individuals with a recombinant breakpoint (dashed lines) near *Vr3*

*b* allele from the susceptible parent ‘Earlygold’, *h* allele from heterozygote individuals, *R* resistant. *S* susceptible, *N* number of recombinant individuals

Five individuals had the nearest recombination to the *Vr3* gene, so determining the *Vr3* region. Two resistant individuals from TxE and 44P17 families and one susceptible individual from 14P16 family showed a recombination between Indel16912 and SNP_16932290, defining the lower limit of the *Vr3* region. Two resistant individuals with a recombination between SSR_17182435 and SNP_17184692, corresponding to families E2T-031-06 and 51P17, defined the upper limit. In this region, spanning ~270 kb, 27 annotated genes (Table 3) were found in the *P. persica* Genome Annotation v2.1^16^ retrieved from the Genome Database for Rosaceae^17^.

**Table 3:** *Vr3* resistance candidate genes to peach powdery mildew

Gene	Position	Predicted function	Function classification
Prupe.2G110900	Pp02:16913576..16914676	Agamous-like MADS-box protein (*Arabidopsis thaliana*)	Metabolism
Prupe.2G111000	Pp02:16920605..16921195	Germin-like protein (*Oryza sativa* subsp. Japonica)	Plant defense
Prupe.2G111100	Pp02:16922483..16922915	n/a	Unknown
Prupe.2G111200	Pp02:16923107..16925825	n/a	Unknown
Prupe.2G111300	Pp02:16926230..16929679	26S protease (*Arabidopsis thaliana*)	Metabolism
Prupe.2G111400	Pp02:16930138..16934333	ABC transporter (*Arabidopsis thaliana*)	Metabolism
Prupe.2G111500	Pp02:16936869..16945304	ABC transporter (*Arabidopsis thaliana*)	Metabolism
Prupe.2G111600	Pp02:16993968..16994329	n/a	Unknown
Prupe.2G111700	Pp02:16996435..17001837	Disease resistance protein RGA2 (*Solanum bulbocastanum*)	Plant defense
Prupe.2G111800	Pp02:17003896..17010678	Putative disease resistance protein RGA3 (*Solanum bulbocastanum*)	Plant defense
Prupe.2G111900	Pp02:17011424..17014545	Hydrolase domain-containing protein Sgpp (*Arabidopsis thaliana*)	Metabolism
Prupe.2G112000	Pp02:17015308..17018250	Endoglucanase 12 (*Arabidopsis thaliana*)	Metabolism
Prupe.2G112100	Pp02:17020262..17023645	Riboflavin biosynthesis protein PYRD (*Arabidopsis thaliana*)	Metabolism
Prupe.2G112200	Pp02:17024102..17036171	DNA replication helicase (*Arabidopsis thaliana*)	Metabolism
Prupe.2G112300	Pp02:17039404..17042658	Zinc ion binding (*Arabidopsis thaliana*)	Metabolism
Prupe.2G112400	Pp02:17049419..17050149	n/a	Unknown
Prupe.2G112500	Pp02:17050202..17050814	n/a	Unknown
Prupe.2G112600	Pp02:17061213..17068962	Protein ECERIFERUM 1 (*Arabidopsis thaliana*)	Structural
Prupe.2G112700	Pp02:17073807..17075686	TMV resistance protein N (*Nicotiana glutinosa*)	Plant defense
Prupe.2G112800	Pp02:17099320..17103427	Protein ECERIFERUM 1 (*Arabidopsis thaliana*)	Structural
Prupe.2G112900	Pp02:17113525..17117895	Protein ECERIFERUM 1 (*Arabidopsis thaliana*)	Structural
Prupe.2G113000	Pp02:17138061..17139410	Protein ECERIFERUM 1 (*Arabidopsis thaliana*)	Structural
Prupe.2G113100	Pp02:17141136..17142354	n/a	Unknown
Prupe.2G113200	Pp02:17142564..17145425	TMV resistance protein N (*Nicotiana glutinosa*)	Plant defense
Prupe.2G113300	Pp02:17151739..17152569	n/a	Unknown
Prupe.2G113400	Pp02:17166049..17166711	RING-H2 finger protein ATL3 (*Arabidopsis thaliana*)	Metabolism
Prupe.2G113500	Pp02:17168568..17172597	Protein ECERIFERUM 1 (*Arabidopsis thaliana*)	Structural

Among the 27 candidate genes, five were annotated as involved in plant defense, an additional five encoding for structural function, ten genes were predicted to be involved in plant metabolism, and seven were annotated as unknown (Table 3). Among the five candidate genes described as involved in plant defense, *Prupe.2G110900* was predicted to function as a germin-like protein. The other four (*Prupe.2G111700*, *Prupe.2G111800*, *Prupe.2G112700*, and *Prupe.2G113200*) were predicted to be plant resistance genes (R genes). Moreover, *Prupe.2G112700* and *Prupe.2G113200* were specifically included in the TIR-NBS-LRR class of plant R genes. Five genes (*Prupe.2G112600*, *Prupe.2G112800*, *Prupe.2G112900*, *Prupe.2G113000*, and *Prupe.2G113500*) were predicted to encode protein Eceriferum 1, involved in epicuticular wax biosynthesis. Finally, of the ten genes predicted to be involved in plant metabolism, three were annotated with hydrolase function (*Prupe.2G111900*, *Prupe.2G112000*, and *Prupe.2G112100*), three related to DNA binding (*Prupe.2G110900*, *Prupe.2G112300*, and *Prupe.2G113400*), three ATP-related genes (*Prupe.2G111300*, *Prupe.2G111400*, and *Prupe.2G111500*) and one predicted as a multifunctional enzyme (*Prupe.2G112200*).

### Variant calling and effect prediction of polymorphisms

A total of 3510 variants including 3073 SNPs, 222 insertions, and 215 deletions were identified (details shown in SM Table 3). These variants were predicted to cause 11,958 effects on the sequences (SM Table 3). Most of them (93.7%) were considered noncoding variants or variants affecting noncoding genes, while 13 (0.11%) were predicted as high-impact variants, 350 (2.9%) as moderate, and 392 (3.28%) as low-impact variants. The 13 high-impact variants producing a disruptive effect on the coded protein were detected in six candidate genes (SM Table 4). Genes annotated as RGA2 resistance protein (*Prupe.2G111700)*, RGA3 resistance protein (*Prupe.2G111800)*, DNA replication helicase (*Prupe.2G112200)*, and three genes with no available annotation (*Prupe.2G111100, Prupe.2G112400* and *Prupe.2G112500*) presented one high-impact variant, whereas genes annotated as Eceriferum 1 (*Prupe.2G113500)* and another with no available annotation presented three and four high-impact variants, respectively.

### Expression analysis of candidate genes

**Fig. 1:**
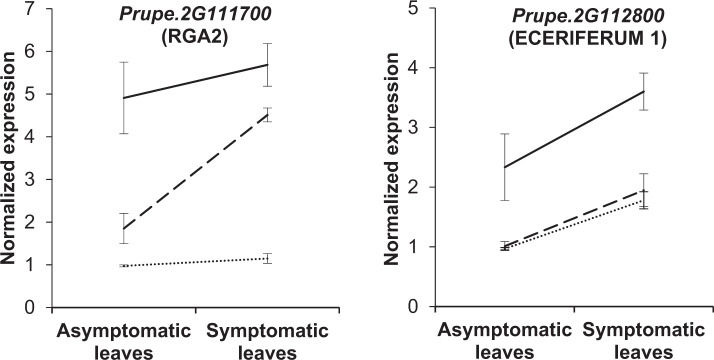
Relative normalized expression of candidate genes with significant differences in symptomatic and asymptomatic leaves (*p* < 0.01). Solid and dashed lines correspond to homozygous (*Vr3Vr3*) and heterozygous (*Vr3vr3*) individuals for the *Vr3* allele from ‘Texas’, respectively. Dotted lines correspond to individuals with *Vr3* peach alleles. Bars indicate standard error of the mean

Relative normalized expression profiles of the 27 candidate genes annotated in the *Vr3* region in this study were analyzed to describe the effect of the infection status and the presence of *Vr3* introgression. One gene, *Prupe.2G111600*, was excluded from the analysis because we could not obtain a regular amplification signal and it was considered inappropriate for qPCR expression analysis. No variants with high or medium impact were detected in this gene. From the 26 candidate genes that could be successively analyzed, Eceriferum 1 (*Prupe.2G112600*) was the only gene with significant interaction (*p* < 0.01) between infection status and the *Vr3* almond allele. Seven candidate genes were found to be significantly differentially expressed for one or both factors (*p* < 0.01). For the infection status factor, three differentially expressed genes were identified, namely Eceriferum 1 (*Prupe.2G113000*), RING-H2 finger protein (*Prupe.2G113400*) and an unknown gene (*Prupe.2G113100*). Their expression increased in all three cases when infection occurred regardless of the *Vr3* allele presence (data not shown). Regarding the allelic status, the genes RGA2 (*Prupe.2G111700*) and Eceriferum 1 (*Prupe.2G112800*) were overexpressed in individuals homozygous (*Vr3Vr3)* and heterozygous (*Vr3vr3)* for *Vr3* (Fig. 1).

In both symptomatic and asymptomatic leaves, the RGA2 annotated gene (*Prupe.2G111700*) had higher relative expression for *Vr3Vr3* individuals compared with *Vr3vr3* individuals: the normalized expression was 4.91 ± 0.84 (mean ± SE) and 1.85 ± 0.36 respectively in asymptomatic leaves, and 5.68 ± 0.50 and 4.51 ± 0.16, in symptomatic leaves. In addition, the normalized expression for susceptible individuals with no *Vr3* almond introgression (1.15 ± 0.11) was significantly lower compared with resistant individuals (*p* < 0.01). Eceriferum 1 (*Prupe.2G112800*) was upregulated for individuals containing the *Vr3* allele, and again gene expression in *Vr3Vr3* differed significantly from heterozygous individuals. Nevertheless, no significant differences were detected among *Vr3vr3* individuals and susceptible individuals not carrying the *Vr3* allele. Finally, genes encoding for Agamous-like MADS-box (*Prupe.2G110900*) and Germin-like protein (*Prupe.2G111000*) were significantly underexpressed in individuals containing the *Vr3* allele (*p* < 0.05), and no interaction between factors was detected.

## Discussion

The PPM resistance gene *Vr3* was located in a 1.8 Mb genomic region of chromosome 2 where 187 genes were annotated in the peach reference genome^12^. In our study, through a fine mapping approach, we narrowed the region down to 270-kb (between Pp02:16,912,811 and Pp02:17,184,692), with 27 genes annotated that were considered as a first set of *Vr3* positional candidate genes. Additional evidence in support of some of these genes being responsible for PPM resistance was gathered through expression analysis and prediction of the effect of variants in the coding sequences of the candidate genes. Among the variants detected in the region, only those predicted to have a high or moderate impact on the protein encoded were considered candidates for the *Vr3* resistance gene. As defined by SNPEff software, high-impact predicted variants were assumed to have disruptive impact in the protein, causing protein truncation or loss of function; and moderate predicted variants might alter protein effectiveness^18^. In the current study, 23 candidate genes were predicted to have a moderate effect on the protein, and six variants also had a high-impact effect.

From the two candidate genes that were differentially overexpressed in resistant individuals, encoding for RGA2 (*Prupe.2G111700*) and Eceriferum 1 (*Prupe.2G112800*), only RGA2 (*Prupe.2G111700*) had a high-impact variant that is producing a stop codon. This gene also presented 35 moderate variants. Regarding gene expression, RGA2 (*Prupe.2G111700*) was the only gene significantly overexpressed in resistant *Vr3Vr3* and *Vr3vr3* individuals compared to susceptible individuals (*vr3vr3*) independently of the infection status. Therefore, RGA2 (*Prupe2.G111700*) was considered our strongest candidate gene for *Vr3*. Moreover, as the expression of RGA2 (*Prupe.2G111700)* did not differ significantly with respect to the infection status, it is assumed to be constitutively expressed, as previously reported for RGA genes involved in fungal resistance in *Rosaceae*, such as for crown rot in octoploid strawberry^19^ and powdery mildew in apple^20^. RGA genes are involved in the recognition and prevention of plant pathogens^21^, with highly conserved amino acid domains that are already known in *P. persica*^22^. *Vr3* has been described as a monogenic resistance gene^12^, and is thought to show completely dominant gene action, being the heterozygous and homozygous plants equally resistant. When comparing the allelic status on the expression of RGA2, homozygous individuals significantly overexpressed *Vr3* as compared to heterozygous individuals despite of the infection status. Conversely, when in heterozygosity, gene expression differed significantly between asymptomatic and symptomatic individuals. This could have implications in the resistance mechanisms and should be borne in mind if this gene is used for breeding purposes.

Another candidate gene differentially overexpressed in resistant individuals was Eceriferum 1 (*Prupe.2G112800*), an ortholog of an *Arabidopsis thaliana* gene related to fungal recognition, based on cuticle wax components^23^. This gene had variants with moderate effect, and was overexpressed only in homozygous individuals containing the *Vr3* almond allele as compared to heterozygous and susceptible individuals. As no significant differences in expression were detected between the susceptible and the *Vr3vr3* individuals, phenotyped as resistant, *Prupe.2G112800* was not considered as a candidate gene for *Vr3*.

Results obtained in this study provide important information to identify a limited number of genes as *Vr3* candidates, responsible for PPM resistance. A validation process through genetic transformation is required, but this is currently difficult due to the recalcitrant character of peach^24^. Another possibility could be the use of a heterologous system such as plum for which an efficient transformation approach has been described^25^, although a limitation of this approach is that species causing powdery mildew in peach differ from that in plum (*P. tridactyla*). This would only be successful if our RGA2 candidate gene conferred broad-spectrum resistance to powdery mildew, as it has been described for the *Pm21* RGA gene in wheat^26^.

Functional validation of these genes is the main bottleneck in *Prunus* due to its recalcitrant regeneration behavior ‘in vitro’^24^. Until efficient peach transformation strategies are available, a feasible alternative to integrate the *Vr3* gene in peach breeding programs could be Marker Assisted Introgression (MAI)^27^. For that, a near-isogenic line carrying a unique introgression from almond containing the *Vr3* gene needs to be developed to cross with the parents from a specific breeding program and then resistant individuals can be selected using the molecular markers described in this work. This strategy is currently in progress in our laboratory to introgress *Vr3* resistant alleles from ‘Texas’ almond into high quality peach commercial cultivars. Finally, we propose to pyramid these lines with other PPM resistance genes such as *Vr2*^6^ to increase PPM resistance durability, and with other peach biotic resistance genes to increase crop sustainability.

## Materials and methods

### Plant material

From 2013 to 2018, several progenies of different generations derived from ‘Texas’ and ‘Earlygold’ crosses were screened for a PPM resistance fine mapping approach: F2 (named TxE, with 111 individuals), BC1 with ‘Earlygold’ as the recurrent parent (named T1E, with 189 individuals) and BC2 also with ‘Earlygold’ (with 51 screened individuals, from E2T-031, E2T-092 and 11P15 families). Other individuals used in the fine mapping approach were obtained from the open pollination of different individuals and families: 218 individuals from ‘MB1.37’ (the ‘Texas’ × ‘Earlygold’ F1 individual used for the construction of the TxE population), 329 individuals from T1E progeny families (named 72P18, 74P18, 84P18, and 93P18), and 217 individuals from BC2 progeny families (including 15P15, 19P15, and 25P15 families). In addition, some recombinant individuals were obtained from crosses with several peach commercial cultivars and individuals from different ‘Texas’ × ‘Earlygold’ generations. This included 81 individuals derived from ‘Nectatop’ crossed with different BC1 and BC2 individuals, All the trees described (Table 1) were planted at IRTA facilities located in Cabrils (41° 31’ 7’ N, 2° 22’ 34’ E), Caldes de Montbui (41° 36’ 47’ N, 2° 10’ 12’ E), Gimenells (41° 39’ 22’ N, 0° 23’ 26’ E), and Mollerussa (41° 37’ 07’ N, 0° 51’ 60’ E). Orchards were not treated with fungicides to allow natural pathogen infections.

Regarding the gene expression analysis, three groups of four individuals from near-isogenic lines coming from open pollination of a BC2 individual were used. A first group contained only one introgression from ‘Texas’ almond in homozygosis in the *Vr3* genomic region, another with one introgression in heterozygosis in the same region, and the final one with no almond introgression in the *Vr3* genomic region, but including an almond introgression in G3. The four individuals of each group were considered as independent biological replicates for each case, and two technical replicates of three young leaves measuring 3–4 cm were sampled from sight-heighted and sun-exposed branches. The samples collected were symptomatic and visually asymptomatic leaves. In addition, the presence of the pathogen in the field was assessed through detection of airborne *P. pannosa* propagules captured with a volumetric spore sampler VPPS 2000 (Lanzoni, Bologna, Italy) and using a specific qPCR-based protocol developed in a previous study (manuscript in preparation).

### Phenotypic evaluation

All recombinant individuals used in this study were phenotyped for PPM susceptibility every year between 2016 and 2019. Each year, PPM was phenotyped twice, first in May or June (corresponding with the developing stage of infection) and then in September (corresponding with the end of infection but with symptoms still noticeable). Young leaves from a minimum of four differently oriented branches were examined for PPM symptoms. A given individual was scored as resistant when total absence of PPM symptoms on leaves was confirmed throughout the monitoring period. In contrast, trees showing PPM symptoms in at least 1 year were considered susceptible. Trees for all the experimental orchards evaluated for PPM resistance were not treated, to ensure infection and serve as positive controls.

### *Vr3* fine mapping

Genomic DNA from the individuals described in Table 1 was extracted from young leaves using a modification of the CTAB protocol^28^ omitting the final RNAse step. DNA quality and concentration were checked and quantified using a DNA spectrophotometer (Nanodrop Technologies, Wilmington, USA).

New markers (Table 2), including SSRs (Simple-Sequence Repeat Markers), InDels (Introgression and Deletion markers), and SNPs (Single Nucleotide Polymorphism) were designed using resequencing data of ‘Texas’, ‘Earlygold’, and ‘MB1.37’. Library preparation and 2 × 100 bp pair-end genome sequencing data were obtained by Serra^29^ using HiSeq2000 sequencer (Illumina Inc.). High-quality 220–480 bp size fragmented DNA was ligated to Illumina paired-end adapters. Adapter removal was done using AdapterRemoval v1.5.2^30^. Only reads with a minimum size of 35 bp and a mean quality of 25 were kept. High quality reads were mapped to the peach reference genome using BWA v0.7.5^31^ with default parameters. The SAM file was converted to BAM using BAMTools v0.1.19^32^ and reads mapping to more than one position or reads from PCR duplication events were excluded from the alignment. Raw Illumina data for ‘Texas’, ‘Earlygold’ and ‘MB1.37’ are available at the European Nucleotide Archive under the accession numbers ERS4540423, ERS3508161, and ERS4540424, respectively.

Polymorphisms in these resequencing data were detected using Integrative Genomics Viewer software^33^. SSRs and InDels (Table 2) were designed from the flanking sequences of the polymorphisms using Primer 3 (http://primer3.ut.ee), v4.1.0^34^ with the default parameters. PCR reactions were in a final volume of 10 μL containing 200 ng of genomic DNA, 1 × NH_4_ reaction buffer, 1.5 mM MgCl_2_, 0.2 mM dNTPs (10 mM), 0.2 μM of each marker and 1 U of BIOTaq (Bioline, London, UK) and HPLC H_2_O to reach the final volume. PCRs were performed in a GeneAmp PCR System 9700 thermal cycler (Applied Biosystems, CA, USA), with the following conditions: initial denaturation at 94 °C for 1 min, 35 cycles of denaturation at 94 °C for 15 s, primer annealing at specific temperature for each primer for 15 s, extension at 72 °C for 30 s, and a final extension at 72 °C for 5 min. For InDels less than 40 bp and for SSRs, forward primers were designed with a generic fluorochrome sequence at the 5′ ends (FAM, VIC, NED, or PET), named ‘tag primers’^35^. PCR reaction conditions for these ‘tag primers’ were the same as described above, with the following modifications: 0.4 μM of each marker and 0.20 μM for each ‘tag’ primer pair. PCR amplifications were with an initial denaturation at 94 °C for 1 min, followed by a total of 60 cycles with the profile: 20 cycles for 15 s at 94 °C, 15 s at 63 °C, and 30 s at 72 °C, followed by 40 cycles for 15 s at 94 °C, 15 s at 54 °C, and 30 s at 72 °C, followed by a final extension step of 5 min at 72 °C. PCR products were added to 12 μL of deionized formamide containing 0.35 μL of GeneScan500 LIZ size standard (Applied Biosystems, CA, USA). The mixture was heated at 94 °C for 3 min and capillary electrophoresed using an ABI Prism 3130xl automated sequencer (Applied Biosystems, CA, USA). GeneMapper v5.0 software (Applied Biosystems) was used for SSR allele sizing. For InDels larger than 40 bp, standard primers were designed flanking the polymorphism and results were observed in ethidium bromide-stained agarose gels (1.8%) under UV light. From all the SSRs and Indels designed, only those showing clear segregation among the parents were kept for the fine mapping approach, avoiding those with preferential amplification for peach alleles or that did not amplify. Otherwise, primers for SNPs detection (Table 3) were designed using the Primer Picker Lite tool from KASPar SNP Genotyping System (KBiosciences, Herts, UK). SNP genotyping was performed by qPCR through a LightCycler 480 device (Roche Diagnostics, Spain) using universal KASPar MasterMix (LGC, Teddington, UK) following the supplier’s technical instructions.

### Prediction of variants effect of candidate genes sequences

Almond and peach resequences of candidate genes defining the *Vr3* region, located between Pp02:16,912,811 and Pp02:17,184,692 physical positions of the *P. persica* v.2.0 reference genome^16^, were compared to predict the variants in the region. Their effect on annotated genes of the region was determined using SNPEffect software 4.3p^18^. Variant effect was defined by the impact on the protein in three categories: (i) high impact, by impairing protein function, i.e., affecting splice-sites or start and stop codons, (ii) moderate impact including nondisruptive variants, and (iii) low impact including synonymous variants.

### Gene expression analysis

#### RNA isolation and cDNA synthesis

The sampled leaves were immediately frozen in liquid nitrogen after collection and RNA was isolated with the Spectrum Plant Total RNA kit (Sigma Aldrich, Munich, Germany), according to the manufacturer’s instructions. RNA concentration and purity were checked with a Nanodrop ND-1000 spectrophotometer. Samples were only further processed for cDNA synthesis, if the 260/280 ratio was between 1.9 and 2.1, and the 260/230 ratio > 2.0^36^. cDNA was synthetized from 1 µg of total RNA for each sample using the PrimeScript RT-PCR Kit (Takara, Otsu-shi, Japan) according to the manufacturer’s instructions.

#### Primer design of candidate genes

Primer pairs (SM Table 5) were designed for the 27 candidate genes defined in the region of interest after *Vr3* fine mapping. Coding DNA sequences of candidate genes were obtained from the Genome Database for Rosaceae^17^. Sequences were analyzed by BLAST alignment for specificity checking^37^, primer pairs suitability (GC content, self-complementarity and dimer formation) was checked using Oligoanalyzer 3.1(Integrated DNA Technologies, URL: https://eu.idtdna.com), and *mfold*^38^ (URL: http://unafold.rna.albany.edu) was used to predict secondary structure formation.

#### qPCR expression analysis

qPCR assays were performed using a Fluidigm 48.48 dynamic array chip on the BioMark HD System Real-Time PCR (Fluidigm, CA, USA). Prior to the high-throughput qPCR, a pre-amplification of the cDNA samples was performed. Diluted (1:3) pre-amplified cDNA samples were loaded according to Fluidigm’s EvaGreen DNA-binding dye protocols. Negative controls were used in the assay to detect possible DNA contamination. Four reference genes used in previous expression analysis were evaluated: actin (*Act*), expansin (*Exp1*)^39^, pre-mRNA splicing factor 7 (*SLU7*)^40^ and translation elongation factor 2 (*TEF2*)^41^. The stability of each reference gene was defined with the SATqPCR statistical analysis tool^42^ based on the geNorm method^43^, considering the lowest gene variability. Considering the use of at least two reference genes, as described in MIQE rules^44^, *Act*, *Exp1*, and *SLU7* were finally chosen for normalizing relative quantities for each candidate gene.

The effects of two factors in the relative expression of candidate genes were considered: (i) disease status, i.e., symptomatic or asymptomatic leaves, and (ii) the presence of the *Vr3* almond alleles, either in homozygosis or heterozygosis. Considering normal distributions and independence of the observations, two-way analysis of variance (ANOVA) was used to assess the independent effect of each of these two factors in normalized expression of all candidate genes. ANOVA tests were performed using the ‘RqPCRAnalysis’ R-package^45^ included in the SATqPCR statistical analysis tool^43^. Orthogonal contrasts were used to detect differences in different levels of the factor describing the disease status. The first contrast was among individuals with *Vr3* introgression (including heterozygous and homozygous individuals) and individuals without the introgression from ‘Texas’, and the second among heterozygous and homozygous individuals. Statistical significance of these tests was set at *α* < 0.01.

## Supplementary information

SM-Vr3-17-07-20

## Data Availability

The data that support the findings of this study are available from the corresponding author upon reasonable request.
