# Supplementary material for: Fine mapping and identification of candidate genes for the peach powdery mildew resistance gene Vr3
Source: Hortic Res. 2020 Nov 1;7:175. doi: 10.1038/s41438-020-00396-9 (PMC7603514; doi:10.1038/s41438-020-00396-9)

**SM Table 1.** Indel and SSR primers used in the fine mapping of *Vr3*. Location is identified from the *P. persica* v.2.0 reference genome^17^.

| **Marker name** | **Forward 5’-3’ sequence** | **Reverse 5’-3’ sequence** | **Marker location (bp)** | **Product size (bp)** |
| --- | --- | --- | --- | --- |
| CPP08188 ^b^ | AAAAGGGGTTTCGGAAGATG | ATGGCATCTCGTCACACTTG | 14,103,569 | 55 |
| Indel14417^a^ | TTTCAATTTGGGTGGTTTGC | CCCAACTCCGAAAAATTCAA | 14,417,443 | 200 |
| Indel15174 ^a^ | ATTCACCTTCATTGGCTTGG | AGGAGATTGTGGTTGGTTCG | 15,174,881 | 81 |
| Indel16614 ^a^ | TTTAACAGGTTGAGATGGTGGT | TGGGGCAGAATCTTTATCCA | 16,614,476 | 35 |
| Indel16724 ^a^ | CCACCAGTGAGCCATCAAC | GGCGTTGACTCCATACGAAA | 16,724,717 | 54 |
| Indel16748 ^a^ | AAGGCTCCCACTGAATGATG | CCTGCAATGTGGTTGACAAT | 16,748,613 | 57 |
| CPP08472 ^b^ | GTCATGCAGACCTCCAATCC | TTGCAGGCTAGGCTAGAGAAA | 16,484,677 | 68 |
| Indel16912 ^a^ | AAGTCTAGTTCCAGCACACC | ACAAGTAAGGGTGTTCATCCAT | 16,912,809 | 76 |
| Indel16949 ^a^ | ACTGTTTATTGTCCTGGATGCA | CTTCAAGCCCGTGACTAGAGT | 16,948,818 | 48 |
| Indel17030 ^a^ | TGACTCTACAGCAGGAAAAGGA | CGATGCTAAAGGTATGGCGG | 17,030,526 | 2 |
| Indel17032 ^a^ | CTCCACAAGTGCAGCCTACA | TGAGAACCACCCTATGATTTTGT | 17,032,695 | 2 |
| Indel17050 ^a^ | GTGCAGGACATCACGGAGAA | TGCGACACACCTGAACGTTA | 17,050,734 | 126 |
| Indel17053 ^a^ | AGACAAGGCACATGACAGCT | GTTGGTTGTTGCTTGAGGACC | 17,053,061 | 2 |
| Indel17061 ^a^ | GGCTGTACTCGCGGATATGA | CAAGAGGAGTCCATGGCCAG | 17,061,201 | 208 |
| Indel17186 ^a^ | AAGGGGGTGTCAATGTCAAG | TGTGGGATACAAATTCCACAAG | 17,186,620 | 46 |
| Indel17181 ^a^ | TGTTTTGATGAAGGCGATCGA | TGGAAGGTTGGAAGGAGCAT | 17,181,256 | 6 |
| CPP17182 ^b^ | TCTCTACTCTTACAGGCGAGC | GGGTTGTGGATGGAAGTAGC | 17,182,435 | 16 |
| CPP17184 ^b^ | GTAGGTTGCAGTTCGACACG | GACACCACAGTACCCACCTT | 17,184,920 | 26 |

^a^ Indel marker. ^b^ SSR marker

**SM Table 2.** SNPs primers used in the fine mapping of *Vr3*. Location is identified from the *P. persica* v.2.0 reference genome^17^.

| **Marker name** | **KASPar forward primers (without tail sequences)** | | **Common reverse primer** | **Location (bp)** |
| --- | --- | --- | --- | --- |
| SNP16932 | AGTGATTCTGCAAAGTGGTGGAGA | GTGATTCTGCAAAGTGGTGGAGG | TGGCACGGCTATCAGGCATAGAAA | 16,932,290 |
| SNP16940 | TGATCGAGAATAAATTCAGATGTTAAAGAATT | ATGATCGAGAATAAATTCAGATGTTAAAGAATA | TGTTGACTTATTGCTCCTTCAGTCTACTT | 16,940,264 |
| SNP17180 | CTCACCAATTTATAAGAGTTTGGTATGTT | CTCACCAATTTATAAGAGTTTGGTATGTC | CGGATGTCCTGCTCCCTTATTGAAT | 17,180,556 |
| SNP17184 | CATATACATCCCAGAGGCCCATATA | ATATACATCCCAGAGGCCCATATG | ACCTCACCACATACTTCCATTGTTTTCTT | 17,184,692 |

**SM Table 3.** Effects of variants detected in the *Vr3* region as predicted by SnpEff.

| **Effect** | **Count** | **Percent** | **Impact** |
| --- | --- | --- | --- |
| frameshift_variant | 7 | 0.06% | High |
| splice_acceptor_variant | 1 | 0.01% | High |
| splice_donor_variant | 2 | 0.02% | High |
| start_lost | 1 | 0.01% | High |
| stop_gained | 2 | 0.02% | High |
| stop_lost | 1 | 0.01% | High |
| inframe_deletion | 6 | 0.05% | Moderate |
| inframe_insertion | 2 | 0.02% | Moderate |
| missense_variant | 342 | 2.85% | Moderate |
| splice_region_variant | 61 | 0.51% | Low |
| synonymous_variant | 305 | 2.54% | Low |
| 3_prime_UTR_variant | 207 | 1.72% | Modifier |
| 5_prime_UTR_premature_start_codon_gain_variant | 34 | 0.28% | Modifier |
| 5_prime_UTR_variant | 222 | 1.85% | Modifier |
| downstream_gene_variant | 3,805 | 31.65% | Modifier |
| intergenic_region | 1,934 | 16.09% | Modifier |
| intron_variant | 1,422 | 11.83% | Modifier |
| upstream_gene_variant | 3,667 | 30.51% | Modifier |

**SM Table 4.** Nucleotide changes of high impact variants detected in the *Vr3* region**.**

**SM Table 5.** Primer sequences used to amplify the candidate genes in the region encompassing *Vr3*.


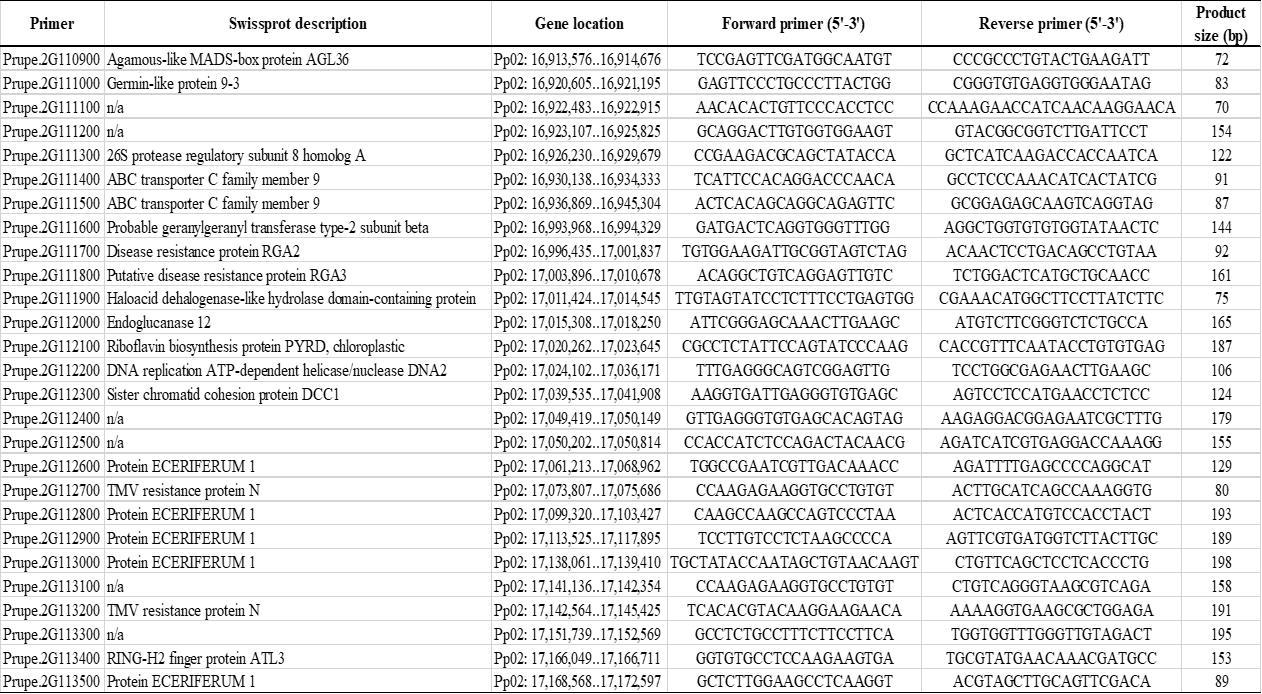

Supplement: Supplementary file 1 — SM-Vr3-17-07-20 [file 41438_2020_396_MOESM1_ESM.docx]
